# Age-Related COVID-19 Influence on Male Fertility

**DOI:** 10.3390/ijms242115742

**Published:** 2023-10-30

**Authors:** Anastasiia D. Shcherbitskaia, Evgeniia M. Komarova, Yulia P. Milyutina, Yanina M. Sagurova, Mariia A. Ishchuk, Anastasiia V. Mikhel, Ksenia V. Ob’edkova, Elena A. Lesik, Alexander M. Gzgzyan, Natalya I. Tapilskaya, Olesya N. Bespalova, Igor Y. Kogan

**Affiliations:** D.O. Ott Research Institute of Obstetrics, Gynecology, and Reproductive Medicine, 199034 St. Petersburg, Russia; evgmkomarova@gmail.com (E.M.K.); milyutina1010@mail.ru (Y.P.M.); yanina.sagurova96@mail.ru (Y.M.S.); mashamazilina@gmail.com (M.A.I.); anastasia.michel39@gmail.com (A.V.M.); obedkova_ks@mail.ru (K.V.O.); lesike@yandex.ru (E.A.L.); agzgzyan@gmail.com (A.M.G.); tapnatalia@yandex.ru (N.I.T.); shiggerra@mail.ru (O.N.B.); ikogan@mail.ru (I.Y.K.)

**Keywords:** nitrotyrosine, total antioxidant capacity, zinc, COVID-19, DNA fragmentation, sperm, male fertility, semen quality, cytokine

## Abstract

The impact of coronavirus on the reproductive health of men attracts the special attention of many researchers. While studies suggest changes in sperm parameters and the possibility of testicular inflammation, further studies are needed to elucidate any potential age-related changes in these findings, which is the purpose of the present study. The semen quality parameters, cytokine concentration, and markers of the pro- and antioxidant system were assessed in 60 men five to seven months after the coronavirus infection and in 77 controls (without a history of coronavirus infection). Additionally, participants were divided into two age groups: less than 35 years and 35 years or older. Notably increased round cell count in ejaculate and reduced sperm hyaluronan binding ability were observed among post-infection patients younger than 35 years. In the same group, a decline in seminal plasma zinc levels and nitrotyrosine in the cell fraction was found. In men over 35 years of age, Coronavirus Disease 2019 (COVID-19) led to increased sperm DNA fragmentation, a decrease in the total antioxidant capacity, and an elevation in the levels of interleukin-1β and interleukin-10. The concentration of interleukin-1β decreased over time following recovery in all affected patients. The data obtained suggest the potential adverse impact of the coronavirus infection on male reproductive health; however, these effects appear to be age-dependent.

## 1. Introduction

The coronavirus infection, caused by the severe acute respiratory syndrome-related coronavirus 2 (SARS-CoV-2), has emerged as one of the most significant global health challenges. The Coronavirus Disease 2019 (COVID-19) pandemic is characterized by a wide range of clinical manifestations, ranging from mild symptoms to severe pneumonia and death [1]. Despite the initial association of the coronavirus with acute respiratory infection, an increasing number of researchers are focusing on its impact on other body systems.

One aspect that has become the subject of active investigation is the potential impact of COVID-19 on male reproductive health. In patients who had COVID-19 3 months before analysis, a decrease in the testicular dimensions and an increase in wave velocity caused by the stiffness of this tissue were found to be significant [2]. Emerging data has indicated alterations in sperm parameters in some men following infection. Reductions in sperm concentration, impairment in motility, and changes in morphology have garnered special attention concerning male reproductive health [3,4,5,6].

The results of different studies provide evidence of a significant impact of COVID-19 on male reproductive function in the short term. However, the precise duration of detrimental impacts on male semen remains uncertain. Given that the human spermatogenic cycle is approximately 72–74 days, there is a prevailing notion that the influence of COVID-19 might potentially persist for a similar duration [7]. Nevertheless, the extended consequences for patients and the potential reversibility of these effects remain areas that require further comprehensive exploration. For instance, in patients recovered from COVID-19 (semen samples were collected around 80 days (IQR, 64–93)), sperm concentration, total sperm count, and total motility were significantly declined when compared with healthy controls [8].

Recent studies have shown that one of the significant reasons for poor sperm quality and male infertility is zinc (Zn) deficiency, which causes an increased level of reactive oxygen species (ROS) and oxidative stress [9]. Our previous study demonstrated that a decrease in Zn in the seminal plasma of patients with COVID-19 was accompanied by an increase in sperm DNA fragmentation and oxidative damage [10]. Some studies have also turned their attention to changes in the levels of antioxidants and oxidative markers in semen and sperm plasma in men after COVID-19. For example, in Iranian patients aged 20–40 years, an increase in ROS and a decrease in superoxide dismutase (SOD) activity were observed throughout the 60 days of the study [11]. Fertile males with a virus infection demonstrated significantly reduced sperm concentration, motility, sperm viability, and total antioxidant capacity (TAC) [6]. Another study shows that the detrimental effects of COVID-19 on sperm properties caused by oxidative stress, as evidenced by induced ROS and malondialdehyde (MDA) levels and decreased TAC, were improved 120 days after diagnosis [5].

Zn also has an anti-inflammatory function by reducing the expression of Tumor Necrosis Factor-α (TNF-α) as an inflammatory cytokine and by increasing the expression of anti-inflammatory cytokines, such as interleukin (IL)-2 and IL-4, in human sperm [9,12]. Additionally, higher Zn levels are associated with better clinical outcomes during coronavirus infection [13]. It is very likely that the coronavirus-induced increase in inflammatory markers not only in the blood [14,15] but also in the seminal plasma of patients, which indicates inflammation in the sperm [11,16], may be caused by a decrease in Zn levels in the body. However, the observed changes in inflammatory markers return to baseline values within three months or more after recovery [16].

Over the past few decades, men in developed countries have increasingly delayed fatherhood [17], and the proportion of men over 35 years of age at the birth of their first child is rapidly increasing [18]. Various studies have reported decreased pregnancy likelihood after sexual activity with men older than 34 years [19], along with an increasing risk of spontaneous abortion with paternal age over 35 years [20]. Therefore, it is essential to monitor the impact of various negative factors, such as COVID-19, on male reproductive health based on age. In young men aged 21 to 35 years, coronavirus infection caused abnormal semen quality, with a significant improvement observed 74–81 days after recovery [21]. Another study observed that in patients aged 30 (IQR, 27–34) years, a coronavirus infection in the last 3 months before sample collection resulted in a reduction in sperm concentration, including the percentage of progressively motile sperm, and an increase in DNA fragmentation. Notably, age (under 30 years or older) did not influence DNA fragmentation, in contrast to the significant impact of a prior history of COVID-19 [22].

Despite this, the impact of COVID-19 on male fertility and possible differences in these effects among various age groups remain poorly understood, which was the main purpose of our research. In this regard, we conducted a study of the effects of a coronavirus infection on standard sperm characteristics, sperm DNA damage, hyaluronan binding ability (HBA), as well as oxidative stress markers, and the levels of pro- and anti-inflammatory cytokines in semen in men of two age groups: those younger than 35 years and those aged 35 years or older.

## 2. Results

### 2.1. Semen Quality Parameters

The comparative analysis confirmed no difference in semen quality parameters, such as the rates of spermatozoa with progressive motility, non-progressive motility, immotile cells, the rate of spermatozoa with normal morphology, and those with head pathology, midpiece, and tail pathologies, in men after COVID-19 when compared to the results of patients without a history of coronavirus infection (Table 1). This was observed in both age groups, those aged less than 35 years and those aged 35 years or more.

We observed an increase (*p* ≤ 0.01) in the number of round cells among men in the <35, COVID+ group compared to the <35, COVID− group. In the same group, a significant reduction (*p* ≤ 0.05) in HBA compared to their age-matched controls was noted. No such changes were observed in groups of patients over 35 years of age.

### 2.2. Sperm DNA Fragmentation

**Figure 1:**
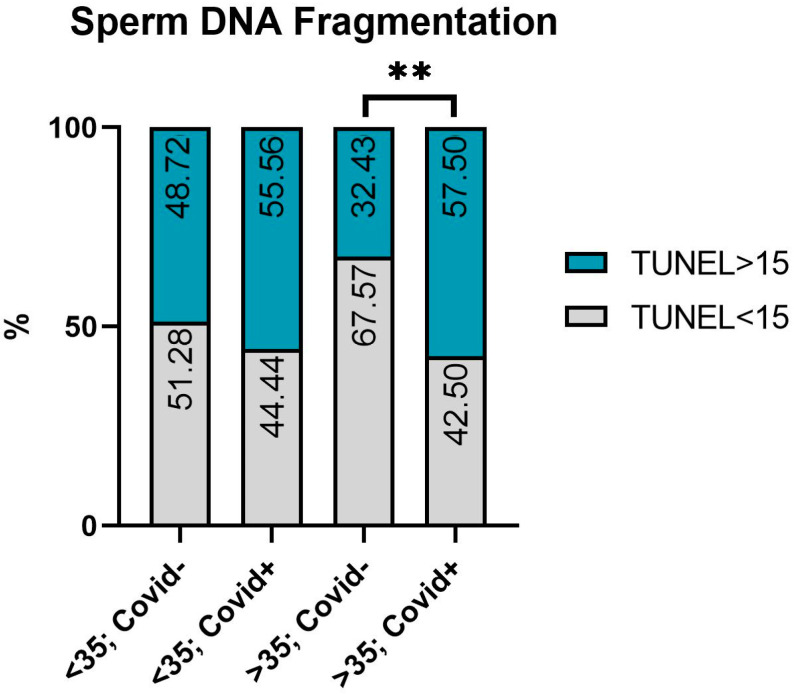
The plot of sample distribution with normal and abnormal TUNEL values in groups aged less than 35 years and 35 years or older after COVID-19 and without it. Pearson’s Chi-square index (χ^2^) was used. **—significant difference between the >35, COVID− and >35, COVID+ groups (*p* ≤ 0.01).

In men aged less than 35 years, no substantial changes in the percentage of TUNEL-positive cells were observed. In the group of men aged 35 or older, the COVID+ patients had a significantly higher (*p* ≤ 0.01) percentage of sperm DNA fragmentation compared to the age-matched patients without a history of SARS-CoV-2 (Figure 1).

### 2.3. Cytokines

**Figure 2:**
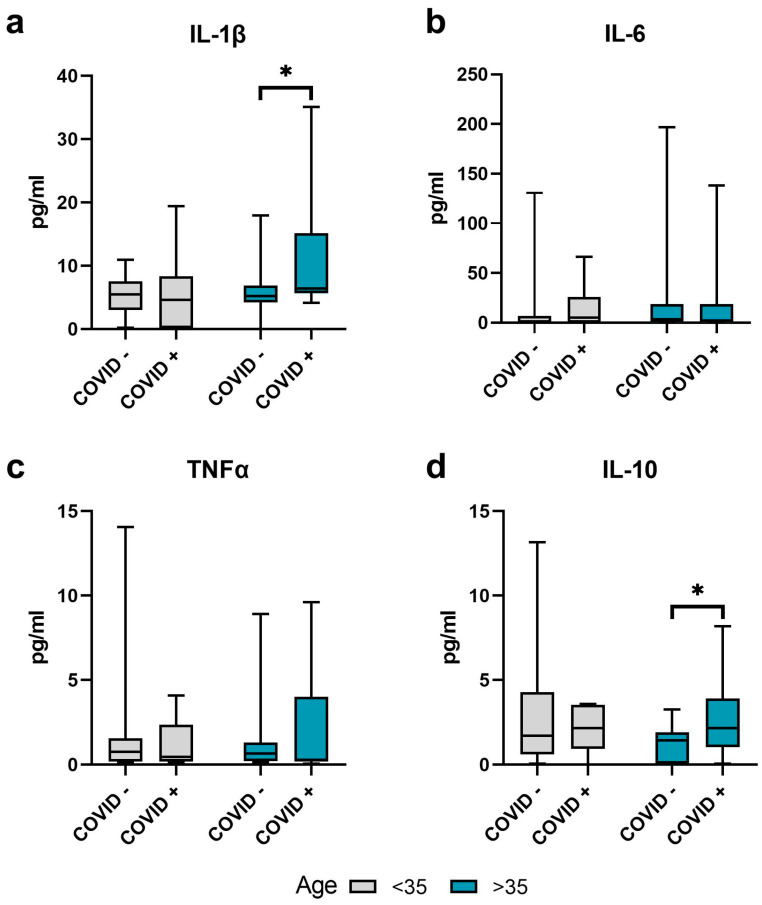
Changes in the seminal markers of inflammation in the COVID+ and control groups, aged less than 35 years and 35 years or older. Concentration of (**a**) IL-1β, (**b**) IL-6, (**c**) TNFα, and (**d**) IL-10 in the seminal plasma. Values are expressed as Me [25, 75%], whiskers—min-max. A significance difference between the >35, COVID− and the >35, COVID+ groups was determined using the Mann–Whitney U test. *—*p* ≤ 0.05.

No significant changes were observed in the concentration of IL-1β, IL-10, IL-6, and TNF-α in COVID+ patients up to 35 years old within their control group (Figure 2).

In men 35 years old or older, increased (*p* ≤ 0.05) levels of IL-1β and IL-10 were observed in the COVID+ group, while IL-6 and TNF-α levels did not differ significantly (Figure 2).

### 2.4. Oxidative Stress Markers

**Figure 3:**
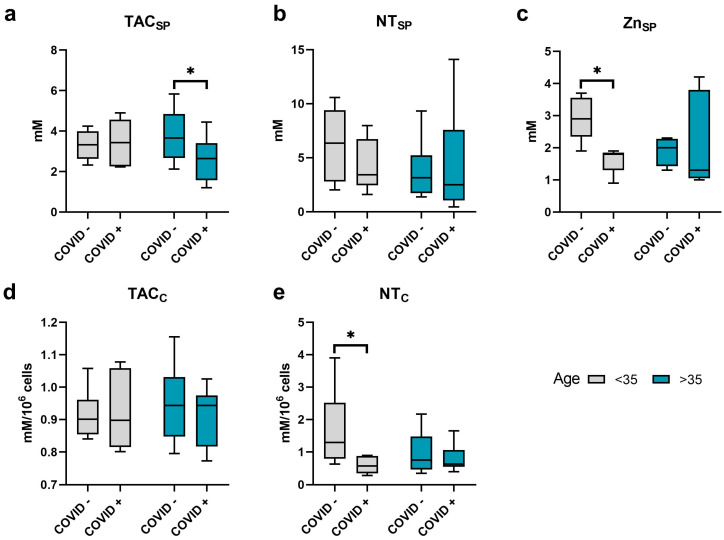
The level of antioxidant components and markers of oxidative modification of proteins in the sperm and seminal plasma of men after COVID-19, depending on their age. The level of total antioxidant capacity (TAC) in seminal plasma (**a**) and sperm (**d**). The level of nitrotyrosine (NT) in seminal plasma (**b**) and sperm (**e**). (**c**) The level of zinc (Zn) in plasma. C (subscript), cellular; SP (subscript), in seminal plasma. Values are expressed as Me [25, 75%], whiskers, min–max. A significant difference between the <35, COVID− and the <35, COVID+ as well as the >35, COVID− and the >35, COVID+ groups, was determined by the Mann–Whitney U test. *—*p* ≤ 0.05.

Among the cohort of men aged less than 35 years, a decrease (*p* ≤ 0.05) in the concentration of Zn in seminal plasma, as well as a decrease (*p* ≤ 0.05) in the content of nitrotyrosine in spermatozoa, was revealed in COVID+ patients (Figure 3). There were no significant changes in the other studied markers in this age group.

At the same time, patients aged 35 years and older showed only a decrease (*p* ≤ 0.05) in total antioxidant capacity (TAC) in seminal plasma after SARS-CoV-2 infection (Figure 3).

### 2.5. Correlations between the Studied Variables of Cytokines

**Figure 4:**
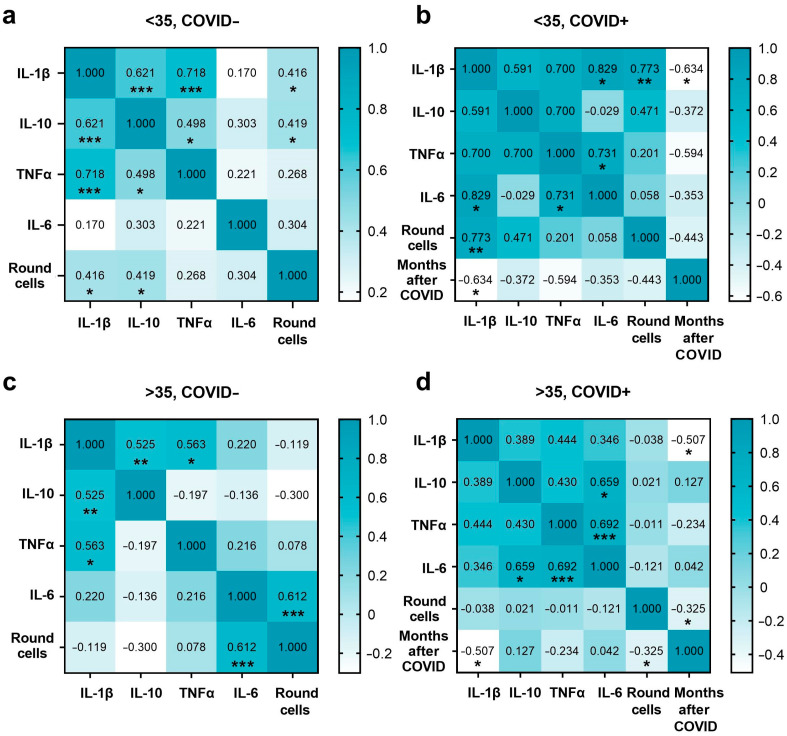
Correlation heat maps of the studied markers in groups (**a**) <35, COVID−; (**b**) <35, COVID+; (**c**) >35, COVID−; (**d**) >35, COVID+. The Spearman correlation coefficient was used to identify the strength and direction of the association between the studied markers. *: *p* ≤ 0.05; **: *p* ≤ 0.01; ***: *p* ≤ 0.001.

We evaluated the association of cytokine levels separately in each group in order to assess the changes between markers depending on various factors. Figure 4 contains the results of Spearman’s analysis for each group.

In the <35, COVID− group, the strongest positive relationship was detected between TNFα and IL-1β in seminal plasma (*p* ≤ 0.001). The increasing level of TNFα was also associated with an increment in IL-10 (*p* ≤ 0.05), while the latter was positively related to seminal plasma IL-1β (*p* ≤ 0.05) and round cell count (*p* ≤ 0.001). A positive association was also demonstrated between IL-1β level and the number of round cells (*p* ≤ 0.05).

In the <35, COVID+ group, seminal plasma IL-1β elevation was associated with an increase in round cell count (*p* ≤ 0.01) and IL-6 level (*p* ≤ 0.05). The Spearman correlation revealed a negative association between the time following COVID-19 infection and the level of IL-1β (*p* ≤ 0.05). The concentration of TNFα was positively correlated with IL-6 (*p* ≤ 0.05).

In the >35, COVID− group, an increase in IL-1β was associated with increasing IL-10 (*p* ≤ 0.01) and TNFα concentration (*p* ≤ 0.05). The IL-6 level in seminal plasma was positively related to the number of round cells (*p* ≤ 0.001).

In the >35, COVID+ group, the concentration of IL-6 was positively related to IL-10 (*p* ≤ 0.05) and TNFα level (*p* ≤ 0.001). In addition, we established a negative association between IL-1β and the number of months after coronavirus infection (*p* ≤ 0.05), while the latter was negatively related to the number of round cells (*p* ≤ 0.05) in this group of patients.

## 3. Discussion

Studies have shown that coronavirus infection significantly affects the quality of sperm, disrupting the morphology of spermatozoa, their mobility, and viability [8,23,24,25,26,27]. Nevertheless, the question of whether COVID-19 has the same impact on the reproductive health of men across different age groups and the duration of these effects over time is still open. It is indicated that changes in sperm morphology were the most prominent abnormalities in sperm characteristics during the infection phase, followed by a significant improvement after recovery from the disease [21]. We assessed standard sperm quality parameters in patients younger and older than 35 years, 5–7 months after recovery from COVID-19. In many studies, the recovery period for sperm parameters is 1 or 2 cycles of spermatogenesis [7,16,28,29]. This may explain why we did not find statistically significant differences in the studied markers. The absence of changes may also be due to the inclusion of patients with mild or moderate coronavirus infection in our sample.

In men from the younger group (<35 years) after recovery from coronavirus infection, we found an increase in the number of round cells in the ejaculate. Since the pool of such cells includes leukocytes and spermatogenic cells, one can also assume the presence of an inflammatory process in the reproductive tract of these patients. We also observed a higher number of round cells in a previous study involving men around 34.5 years of age who exhibited an abnormal level of DNA fragmentation after recovering from COVID-19 [10]. Our findings align with studies conducted by other researchers, which have similarly reported elevated leukocyte counts in the ejaculates of men who have recovered from COVID-19, but their age ranged from 19 to 43 years [4].

According to recent reports, various levels of cytokines are typically found in seminal plasma within normal ranges [30]. However, even 2–3 months after recovering from COVID-19, alterations in the levels of both pro-inflammatory cytokines, such as IL-1β, IL-6, TNFα, and the anti-inflammatory cytokine IL-10, have been observed in semen [11,16]. Additionally, Yu et al. have suggested that the cytokine storm in COVID-19 patients is associated with enhanced levels of 13 specific cytokines (including IL-1, IL-6, IL-10, and TNFα), potentially affecting male fertility through damage to testicular cells and impairment of the immune microenvironment [31]. Furthermore, diverse changes in IL-1β, IL-6, TNFα, and IL-10 have been associated with various spermatogenic disorders [32,33,34]. It is believed that altered levels of cytokines could impact Sertoli cell functions [35,36]. Therefore, in this study, we analyzed the concentrations of IL-1β, IL-6, TNFα, and IL-10 in the seminal plasma of our patients.

A significant increase in cytokine levels in the semen of patients with COVID-19 has been noted in several studies. Higher levels of IL-1β, TNF, and interferon-γ have been found in the semen of men recently recovered from mild and/or severe coronavirus infection [37]. Immune factors such as IL-6, TNFα, and monocyte chemoattractant protein-1 were increased in the semen of COVID-positive patients [37]. In the prospective, longitudinal cohort study, excessive production of IL-1β, IL-6, IL-8, transforming growth factor-β, interferon-α, and interferon-γ was observed in the semen of patients with COVID-19 during a 60-day research period [11]. In our study, among men in the older age group (>35 years), we found an increase in IL-1β and IL-10 after coronavirus infection. Elevation of IL-1β indicates an inflammatory process in the reproductive tract of these patients, as this cytokine is considered a pro-inflammatory molecule. Meanwhile, IL-10 is recognized as an anti-inflammatory cytokine [38]. In this regard, the question arises whether the increase in IL-10 is a compensatory reaction of the organism or, depending on the conditions, IL-10 can perform pro-inflammatory functions. Other researchers also observed a similar increase in the concentration of this cytokine in patients with COVID-19 [11].

We did not observe a significant increase in semen cytokines during the first few months after the SARS-CoV-2 infection. It is highly likely that this is due to the limited number of patients in our sample who had COVID-19 1–3 months before the test. However, our correlation analysis results demonstrate that, over time, the level of IL-1β decreases in both age groups of affected men, which is consistent with the data of other researchers [16].

An increased concentration of seminal cytokines and leukocytes may cause sperm abnormalities by activating the development of oxidative stress. In the group of COVID+ patients under 35 years of age, a decrease in the Zn level was found. Zn is considered a non-enzymatic antioxidant, as it serves as a cofactor of antioxidant defense enzymes. Consequently, in these young men, the protection of germ cells from free radicals may be impaired. Spermatozoa are highly susceptible to free radicals because their cell membranes contain a substantial amount of polyunsaturated fatty acids [39]. While we did not assess the level of lipid oxidative damage in this study, another study reported an increase in the MDA level in the semen of patients who had a coronavirus infection [5,40]. Together with a decrease in the seminal plasma Zn level in these patients, a decline in the NT content was observed in their spermatozoa. In a previous study, we noted a similar decrease in NT in the seminal plasma of men who had COVID-19 and had increased DNA fragmentation [10]. Given the absence of changes in TAC in young men who have had a coronavirus infection, the question of whether the NT level decrease is a compensatory reaction or a disruption of the functional state of spermatozoa, as some researchers suggest [41], remains open and requires more detailed studies. Nevertheless, the data obtained suggest that NT is undoubtedly a marker deserving attention from clinicians managing patients after COVID-19.

After COVID-19 in men of the older study group, we noted a decrease in TAC in seminal plasma, where the largest part of the antioxidant defense components of sperm is located since the cytoplasm is almost completely reduced in spermatozoa. Our data align with other research where a significant inverse correlation was observed between TAC and COVID-19 and the sperm DNA fragmentation index was positively associated with the virus [6]. Moreover, TAC levels have been shown to be reversible over time after recovery [5,10]. TAC consists of many components of both enzymatic and non-enzymatic defense mechanisms. In this study, we did not investigate enzyme activity. However, based on limited literature data, it can be assumed that COVID-19 may affect the activity of SOD [11]. Nonetheless, in our previous study, no changes were observed in either this enzyme or catalase in the semen of patients with varying DNA fragmentation indexes after COVID-19 [10].

The development of inflammation and oxidative stress negatively affects the functional state of spermatozoa. Studies have noted an increase in DNA fragmentation in COVID-positive patients [4,5,6,11]. However, the violation of sperm DNA integrity was shown to be reversible, and this parameter is restored after several cycles of spermatogenesis [27]. At the same time, we did not find any studies that took into account different age groups of patients. In men under the age of 35 who had recovered from a coronavirus infection, we observed no significant changes in DNA fragmentation levels, in contrast to the findings of other researchers [21,22]. However, in our sample of older COVID+ patients (>35 years), the proportion of men with increased DNA fragmentation was higher compared to their peers who have not been infected. Studies have shown that a spermatozoon, even with fragmented DNA, can realize its ability to fertilize [42]. The absence of changes in HBA scores in COVID+ patients aged 35 and older also confirms the possibility of sperm transferring genetic material with impaired integrity to the oocyte. It has been established that the oocyte can repair single-strand damage, while double-strand breaks are irreversible [43]. It is assumed that sperm DNA damage that exceeds the repair ability of the oocyte, or the inability of the oocyte to repair DNA breaks, negatively affects the developmental potential of the embryo and the offspring’s health [44,45].

Hyaluronic acid plays an important role in the process of binding sperm to the oocyte, as it provides intercellular adhesion and is involved in the formation of the oocyte-cumulus complex. There is some evidence that COVID-19 reduces HBA scores in men aged 19 to 52 years (mean age 32–33 years) who had an asymptomatic or mild coronavirus infection [46]. However, the impact of COVID-19 on this process is still not well understood. In our study, the effects of COVID-19 on HBA scores were most marked in the younger male group. The mechanisms of this decrease in the ability to bind hyaluronan acid are not yet clear, but it can be assumed that this may be due to an impairment in the expression of hyaluronic acid receptors on the surface of the spermatozoa. At the same time, in immature spermatozoa with elevated cytoplasmic retention, lower expression of heat shock protein family A (Hsp70) member 2 (HspA2) has been shown [47]. Spermatozoa that are not bound to hyaluronan acid have reduced amounts of HspA2, which is essential for meiosis; thus, they cannot undergo cytoplasmic membrane remodeling and consequently are unable to fertilize the oocyte [48]. Thus, it can be assumed that sperm maturation is significantly impaired in the young COVID+ patients, which is also confirmed by our data on an increase in the number of round cells, including immature germ cells and leucocytes.

Our study has a number of limitations, one of which is a relatively small sample size. We were also unable to retrospectively evaluate patients with COVID-19. Accordingly, there is no baseline semen quality that could reveal spermatogenic defects prior to the onset of the disease. However, we attempted to overcome these limitations with strict exclusion criteria and robust statistical techniques. Future investigations with larger patient cohorts in a variety of age groups are necessary to validate our findings.

## 4. Materials and Methods

### 4.1. Patients

This study was conducted by the Department of Reproductive Medicine in the D.O. Ott Research Institute of Obstetrics, Gynecology, and Reproductive medicine and approved by the Ethics Committee. From October 2020 to January 2023, we recruited 433 participants who voluntarily consented to the use of their biomaterial and questionnaire data for research purposes, provided semen samples, and had records of COVID-19 testing. Criteria for exclusion of participants from the study included conditions such as azoospermia, varicocele, cryptorchidism, prostatitis, urinary tract infections, genital trauma, testicular torsion, groin or genital surgery, sexually transmitted diseases, chronic diseases, severe systemic disorders, antioxidant and vitamin supplementation, use of drugs such as habitual drugs, and occupational and environmental exposure to potential reproductive toxins. A total of 137 male participants (aged 26–54 years) were included to observe the effect of COVID-19 on male fertility. 60 patients were diagnosed with COVID-19 and recovered within 7 (IQR: 4–11) months (COVID+), while the COVID− (or control group) consisted of 77 healthy men who had not previously been diagnosed with COVID-19. In each group, patients were divided into two age subgroups: “<35” (age less than 35 years) and “>35” (age 35 and older).

As a prospective cohort study, the calculation of the optimal sample size required for our research was carried out using the G*Power software (version 3.1.9.6) [49]. This study used a double-blind method. The researchers who performed the measurements and analyzed the data did not know which group the patients belonged to because they were not involved in determining the experimental groups.

### 4.2. Semen Analysis and Preparation

The semen samples were collected between 3 and 5 days of sexual abstinence by masturbation, and the standard semen analysis was carried out according to the World Health Organization criteria of 2010 [50]. We assessed the rate of spermatozoa with progressive motility, non-progressive motility, immotile cells, the rate of spermatozoa with normal morphology, head pathology, midpiece and tail pathologies, round cell count, total sperm volume, and sperm concentration. The volume was measured in a centrifuge tube (Sarstedt, Newton, MA, USA). Sperm concentration was assessed in 10 squares of Makler’s Sperm Counting Chamber using a light microscope (Zeiss Axiostar plus, Carl Zeiss, Oberkochen, Baden-Württemberg, Germany). The total number of spermatozoa per ejaculate was obtained by multiplying the sperm concentration by the volume of the whole ejaculate.

The demographic characteristics of the patients and some semen parameters are detailed in Table 2.

After semen analysis, each fresh semen sample was divided into two aliquots. One of them was immediately served for TUNEL staining, and the other was centrifuged for 15 min at 3000× *g*, +4 °C, to obtain a sperm pellet and seminal plasma. The sperm cell pellet was washed three times in phosphate buffered saline (PBS, pH 7.4, Sigma-Aldrich, St. Louis, MO, USA) and then centrifuged for 15 min at 3000× *g*, +4 °C. After that, 20 million sperm cells were homogenized in radioimmunoprecipitation assay buffer (RIPA lysis buffer, Sigma-Aldrich, St. Louis, MO, USA) and centrifuged for 15 min at 10,000× *g* at +4 °C. The seminal plasma and the supernatant from the sperm cell pellet were frozen at −80 °C until oxidative stress parameter analysis.

### 4.3. Sperm DNA Fragmentation

Sperm DNA fragmentation was assessed by a terminal deoxynucleotidyl transferase-mediated dUTP nick-end labeling assay (TUNEL) using a commercial kit (Roche Diagnostics, Mannheim, Germany) according to the manufacturer’s instructions. Cell fixation was carried out by a methanol/acetic acid mixture (3 volumes/1 volume). Cell suspensions were spread out over slides and dried for 24 h at room temperature. The fixed spermatozoa on the slides were washed two times for 5 min with 1× PBS and additionally fixed in a solution of 4% parafolmaldehyde for 10 min. Then, sperm cells were permeabilized with 0.1% Triton X-100 (Sigma-Aldrich, St. Louis, MO, USA) containing 0.1% sodium citrate (Sigma-Aldrich, St. Louis, MO, USA) for 15 min on ice and rinsed twice with 1× PBS. The samples were incubated in a dark, moist chamber at 37 °C for 1 h with the TUNEL reaction mixture. Slides were washed two times with 1× PBS, and cover slips were mounted using mounting medium (DAPI, H-1200, Vectashield Vector Labs, Newark, CA, USA). The percentage of spermatozoa with fragmented DNA was determined by direct observation of 2000 spermatozoa per sample with a fluorescence microscope (Leica DM 5000B, Wetzlar, Germany) under magnification ×1000. Bright green fluorescence within spermatozoa (wavelength: 488 nm) characterized damaged (fragmented) DNA, while blue (DAPI) fluorescence was indicative of normal DNA structure. The percentage of sperm with DNA fragmentation from the total number of sperm analyzed was calculated. The cut-off value of 15 for TUNEL was determined at our laboratory based on 20 healthy sperm donor evaluations after proven clinical pregnancy.

### 4.4. Hyaluronan Binding Assay

Sperm-Hyaluronan Binding Assay (HBA test) was performed following the manufacturer’s instructions (CooperSurgical, Trumbull, CT, USA). 10 µL of well-mixed semen was added to the assay chamber, and the coverslip was immediately installed. After incubation for 10 min, the unbound motile sperm and bound motile sperm were counted. For the HBA test, 200 motile sperm were counted. The percentage of hyaluronan-binding spermatozoa was calculated by dividing the bound motile sperm by the sum of bound and unbound motile sperm counted in the same squares and then multiplying by 100.

### 4.5. Assessment of Cytokines

IL-1β, IL-10, IL-6, and TNFα concentrations in seminal plasma were analyzed using enzyme-linked immunosorbent assay (ELISA)-specific kits carefully following the manufacturer’s instructions. IL-1β and IL-10 were quantitated by the respective ELISA kits (Interleukin-1β-ELISA-BEST, A-8766, Interleukin-10-ELISA-BEST, A-8774, Vector-Best, Novosibirsk, Russia). The inter- and intra-assay coefficients of variation (CV) were 6.0% and 6.0%, respectively. The standard concentrations were 0–100 pg/mL for IL-1β and 0–200 pg/mL for IL-10. IL-6 and TNFα were assayed by the respective ELISA kits (IL-6, TNF-alpha, Cytokine Ltd., St. Petersburg, Russia). The inter- and intra-assay CV was <10.0%. The standard curve range was 0–125 pg/mL for IL-6 and 0–62.5 pg/mL for TNFα. The amounts of these cytokines were measured through optical densitometry at 450 nm in the microplate reader (ELx800, BioTek Instruments, Winooski, VT, USA). Samples were analyzed in triplicate, and the results were expressed as pg/mL.

### 4.6. Assessment of Total Antioxidant Capacity

To evaluate the TAC present in seminal plasma and spermatozoa, the Trolox equivalent antioxidant capacity method was employed. The assay was conducted spectrophotometrically with an Antioxidant Assay Kit (709001, Cayman Chemical Co., Ann Arbor, MI, USA). Seminal plasma was diluted with assay buffer before analysis. The inhibition of 2,2’-azino-bis(3-ethylbenthiazoline)-6-sulphonic acid oxidation by the ferryl myoglobin-H_2_O_2_ system determined the TAC. The absorbance at 405 nm was measured using a plate reader (EL 800, BioTek Instruments, USA) and expressed as millimolar Trolox equivalents (mmol/L for seminal plasma and mmol/10^6^ cells for spermatozoa). Measurements were taken three times for both standards and samples.

### 4.7. Assessment of Nitrotyrosine Content

The quantity of nitrotyrosine in seminal plasma and spermatozoa was evaluated by a sandwich enzyme-linked immunosorbent assay with the help of Hycult Biotech’s Nitrotyrosine ELISA Kit (HK501, Hycult Biotech, Uden, The Netherlands). The manufacturer’s instructions were followed, and the results were read off at 450 nm. The test was conducted three times for both standards and samples.

### 4.8. Zinc Analysis

The DIALAB diagnostic reagent kit (Wiener Neudorf, Austria) was used to assess the Zn concentration in seminal plasma. The absorbance was determined at 560 nm when a red chelate complex of 2-(5-Bromo-2-pyridylazo)-5-(N-propyl-N-sulfo-propylamino)-phenol was formed. This procedure was carried out using an automatic immunochemical analyzer, UniCel DxI 600 (Beckman Coulter Life Science, Indianapolis, IN, USA).

### 4.9. Statistical Analysis

The data were tested for homogeneity of dispersions using Levene’s test, whereas the normality of distribution was assessed by the Shapiro-Wilk test. Data whose distribution did not follow a normal law were compared using a nonparametric statistical method—the Mann–Whitney U test. Data were reported as box-and-whisker plots displaying medians (25th, 75th percentile) and the maximum and minimum as “whiskers”. The Spearman correlation coefficient (ρ) was used to evaluate the strength and direction of the relationship between the examined indicators. When evaluating markers on a nominal scale, Pearson’s Chi-square index (χ^2^) was used. Statistical analysis was performed using the STATISTICA 10.0 software. Values of *p* ≤ 0.05 were considered statistically significant.

## 5. Conclusions

While more research is still required to fully understand the impact of COVID-19 on male fertility parameters, the available evidence points to the negative consequences of this infectious disease. When planning a pregnancy, special attention should be given to men with a history of COVID-19, particularly with regard to their age. Our study reveals that the effects of a previous coronavirus infection differ across age groups. We observed that in patients under the age of 35 years, coronavirus infection led to an increase in the number of round cells and a decrease in Zn levels in the ejaculate. Simultaneously, there was a decrease in HBA and NT in the cell fraction. In men aged 35 years and older, we noted an increase in the proportion of sperm with fragmented DNA, higher concentrations of cytokines such as IL-1β and IL-10, and a decrease in the antioxidant defense of seminal plasma after COVID-19. These findings emphasize the importance of age-related considerations when evaluating the effects of COVID-19 on male fertility.

## Figures and Tables

**Table 1:** Changes in the semen quality parameters in the groups of men. The values are presented as the median and interquartile range or n (%).

Parameters	<35, COVID−	<35, COVID+	>35, COVID−	>35, COVID+
**Progressive** **motility (PR, %)**	61.50 [49.00–70.00]	57.00 [50.00–62.00]	63.00 [55.00–72.00]	61.00 [50.00–69.00]
**Nonprogressive** **motility (NP, %)**	7.00 [6.00–11.00]	8.00 [6.00–12.00]	8.00 [5.00–9.00]	7.00 [6.00–10.00]
**Immotility** **(IM, %)**	30.00 [21.00–39.00]	31.00 [28.00–42.00]	28.00 [21.00–41.00]	30.00 [22.00–42.00]
**Round cells** **(10^6^ cells/mL)**	0.30 [0.10–0.60]	1.00 [0.70–2.00] **	0.40 [0.00–1.00]	0.40 [0.10–4.00]
**Normal** **morphology (%)**	3.50 [2.00–5.00]	3.00 [1.00–4.00]	3.00 [2.00–5.00]	3 [1.00–4.00]
**Head defects (%)**	91.00 [87.00–93.00]	91.00 [89.00–94.00]	90.00 [87.00–94.00]	92.00 [88.00–94.00]
**Midpiece defects (%)**	1.00 [0.00–1.00]	1.00 [0.00–1.00]	1.00 [0.00–1.50]	1.00 [0.00–1.00]
**Tail defects (%)**	0.00 [0.00–1.00]	1.00 [0.00–1.00]	0.00 [0.00–1.00]	0.00 [0.00–1.00]
**HBA (%)**	89.50 [88.00–93.00]	70.00 [50.00–92.00] *	83.00 [25.00–88.00]	89.00 [71.00–90.00]

*—significant difference between the <35, COVID− and <35, COVID+ groups (*p* ≤ 0.05); **: significant difference between the <35, COVID− and <35, COVID+ groups (*p* ≤ 0.01).

**Table 2:** Demographic and clinical characteristics of patients. The values are presented as median and interquartile range or as n (%).

Parameters	<35, COVID−	<35, COVID+	>35, COVID−	>35, COVID+
**n**	39	20	38	40
**Age (years)**	31 [29–33]	33 [21–34]	38.5 [37–40]	37.5 [36–40]
**BMI**	25 [24–27]	25 [23–27]	26 [24–30]	26 [23–28]
**Months after COVID**	NA	7 [4–12]	NA	5 [3–11]
**Paternity (%)**	11	5	35	44
**Smoking (%)**	30	32	32	28
**Alcohol consumption (%)**	22	32	27	38
**Volume (mL)**	4 [3–5]	3.5 [3–4.75]	3.25 [2.50–4.00]	3.25 [3–4.75]
**Sperm concentration** **(mln/mL)**	82 [61–136]	70 [50–110]	112.5 [69.00–171]	104.5 [69–157]

NA—not applicable.

## Data Availability

The data that support the findings of this study are available on request from the corresponding author. The data are not publicly available due to privacy or ethical restrictions.
